# Immune suppression in gliomas

**DOI:** 10.1007/s11060-020-03483-y

**Published:** 2020-06-15

**Authors:** Matthew M. Grabowski, Eric W. Sankey, Katherine J. Ryan, Pakawat Chongsathidkiet, Selena J. Lorrey, Daniel S. Wilkinson, Peter E. Fecci

**Affiliations:** Duke Brain Tumor Immunotherapy Program, Duke University Medical Center, 303 Research Drive, 220 Sands Bldg, Durham, NC 27710 USA

**Keywords:** Glioblastoma, Gbm, Glioma, Immune suppression, Immunosuppression, Immunotherapy

## Abstract

**Introduction:**

The overall survival in patients with gliomas has not significantly increased in the modern era, despite advances such as immunotherapy. This is in part due to their notorious ability to suppress local and systemic immune responses, severely restricting treatment efficacy.

**Methods:**

We have reviewed the preclinical and clinical evidence for immunosuppression seen throughout the disease process in gliomas. This review aims to discuss the various ways that brain tumors, and gliomas in particular, co-opt the body’s immune system to evade detection and ensure tumor survival and proliferation.

**Results:**

A multitude of mechanisms are discussed by which neoplastic cells evade detection and destruction by the immune system. These include tumor-induced T-cell and NK cell dysfunction, regulatory T-cell and myeloid-derived suppressor cell expansion, M2 phenotypic transformation in glioma-associated macrophages/microglia, upregulation of immunosuppressive glioma cell surface factors and cytokines, tumor microenvironment hypoxia, and iatrogenic sequelae of immunosuppressive treatments.

**Conclusions:**

Gliomas create a profoundly immunosuppressive environment, both locally within the tumor and systemically. Future research should aim to address these immunosuppressive mechanisms in the effort to generate treatment options with meaningful survival benefits for this patient population.

## Introduction

The overall survival in patients with gliomas has not improved significantly over the past decades, despite aggressive treatments [1]. Recent research within the field has shown an increased emphasis on understanding the complex relationship between the immune system and these deadly central nervous system (CNS) tumors. The present findings have significant implications not only from a research standpoint, but also in the daily management and treatment of glioma patients. This review aims to discuss the various ways that brain tumors, and gliomas in specific, co-opt the body’s immune system to evade detection and ensure their proliferation and survival.

## Immune cell dysfunction

### Lymphocyte dysfunction

#### T-cells

**Fig. 1:**
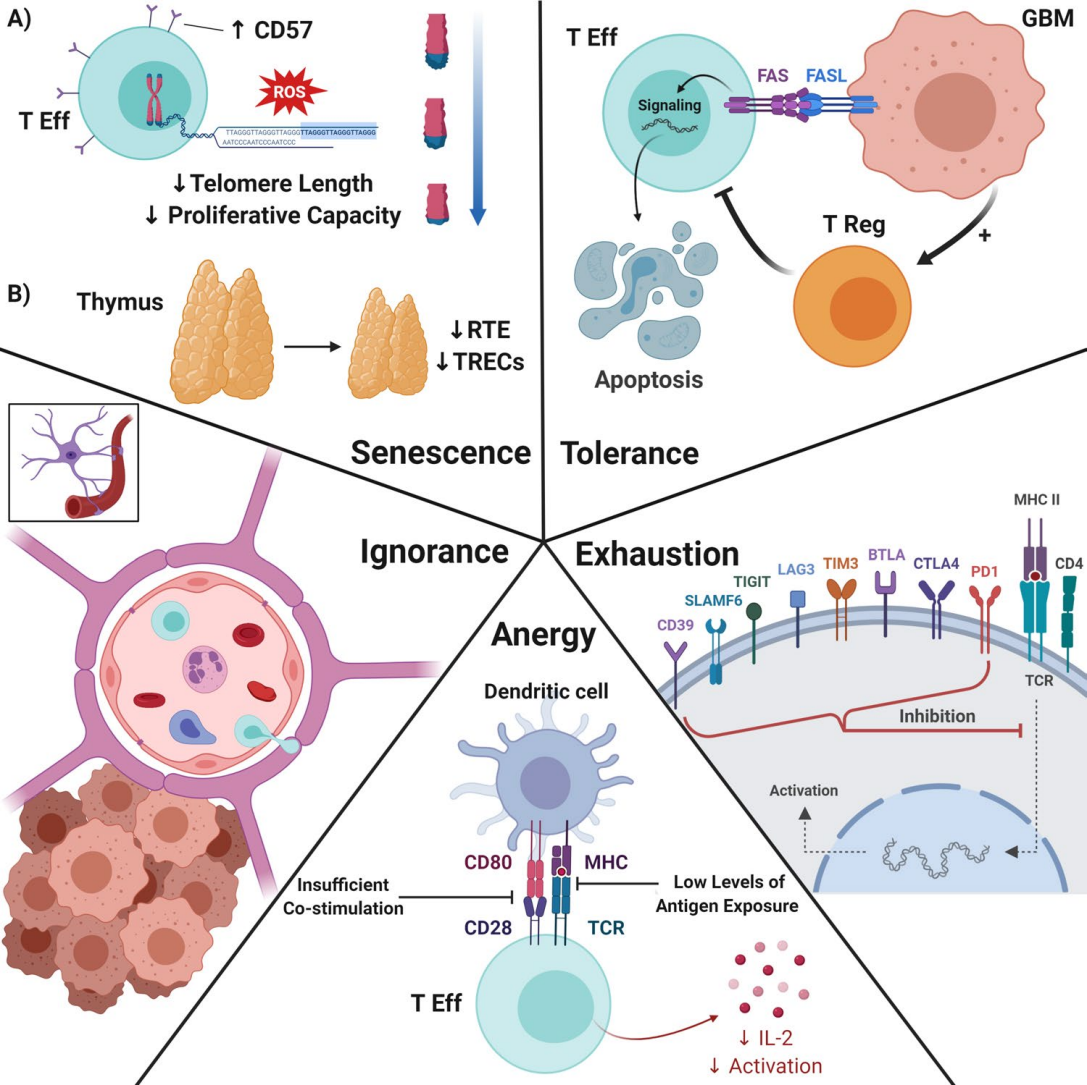
Five domains of T-cell dysfunction. Clockwise from top left—Senescence: **a** Repetitive T-cell proliferation/activation and DNA damage events cause telomere shortening, decreasing the proliferative capacity of effector T-cells. **b** Thymic involution develops prematurely in patients with GBM, reducing T-cell output from the thymus. Tolerance: Gliomas induce T-cell apotosis via the FasL-Fas pathway, as well as generate proliferation of Tregs, which have suppressive effects on effector T-cells. Exhaustion: After repeated exposure under suboptimal conditions, T-cells end up expressing inhibitory immune checkpoints, with the major ones shown here. The degree of exhaustion is correlated with expression of specific checkpoints. Anergy: T-cell anergy can be caused by two broad mechanisms: insufficient co-stimulation leading to clonal anergy and impairment of T-cell activation, and continuous low level antigen exposure, leading to adaptive tolerance and reduced T-cell proliferation. Ignorance: T-cell ignorance is the result of fully functional T-cells that are prevented from antigen exposure by anatomical barriers or insufficient antigen expression levels, such as is the case with the blood brain barrier and T-cell sequestration. *T Eff* effector T-cell, *ROS* reactive oxygen species, *RTE* recent thymic emigrants *TRECs* T-cell receptor excision circles, *T reg* regulatory T-cell, *MHC* major histocompatibility complex, *TCR* T-cell receptor. Created with BioRender.com

High grade gliomas (HGG) are one of the most immunosuppressive solid tumors despite rare metastasis outside the CNS [2]. The ability to cause severe, systemic T-cell deficits is one of the most prominent and earliest reported immune-related effects of HGGs (1). T-cell dysfunction in HGG (and glioblastoma [GBM] in specific) can be molecularly categorized into 5 domains: senescence, tolerance, anergy, exhaustion, and ignorance (Fig. 1) [3].

T-cell senescence is thought to be caused by telomere shortening from repetitive T-cell proliferation/activation and DNA damage events, such as exposure to reactive oxygen species (ROS) [4]. Proposed signature markers of T-cell senescence are upregulation of CD57, an indicator for T-cell terminal differentiation, as well as loss of CD27 and CD28, which are costimulatory markers [5, 6]. These phenotypes correlate well with telomere shortening and telomerase activity loss. In GBM, T-cell senescence phenotype suggests poor prognosis, as GBM patients with higher level of CD4^+^CD28^−^CD57^+^ T-cells have shorter overall survival [7]. Additionally, thymic involution develops prematurely in patients with GBM. This phenomenon results in a reduced output of naïve T-cells (known as recent thymic emigrants [RTE]) from the thymus [8]. Lower RTE, as measured by lower T-cell receptor excision circles (TREC, indicating thymic senescence), was also shown to correlate with poor clinical outcomes in GBM patients [9].

In the normal physiologic state, the body prevents autoimmunity through T-cell tolerance [10]. Central tolerance, mediated by negative selection in the thymus, is imperfect, with the chance for self-antigen reactivity. Therefore, peripheral tolerance outside the thymus serves as an additional safety net against autoimmunity. Peripheral T-cell tolerance is normally comprised of peripheral deletion and suppression by regulatory T-cells (Tregs). However this mechanism is hijacked by tumors, preventing an effective antitumor immune response [11]. T-cell apoptosis, mediated by the FasL-Fas pathway, has been described as a mechanism to delete T-cells in several types of cancer, including GBM [12]. The role that Tregs play in this peripheral T-cell tolerance in HGG will be discussed in a subsequent section.

T-cell anergy was originally used to describe the lack of type IV hypersensitivity response found in GBM patients who failed to react to recall antigen [13]. However, the term anergy now covers two separate entities: clonal/in vitro anergy and adaptive tolerance/in vivo anergy [13]. Clonal anergy is caused by insufficient co-stimulation, leading to defective RAS/MAPK activation and AP-1 transcription, which impairs T-cell activation [14]. Alternatively, adaptive tolerance arises from continuous low levels of antigen exposure, which leads to NF-κB impairment, low IL-2 production, and reduced T-cell proliferation [14]. While each entity represents different T-cell molecular states, both are present in GBM patients and contribute to global T-cell dysfunction.

Classically described in chronic viral infection, T-cell exhaustion occurs after repeated antigen exposure under suboptimal conditions. This results in activation of a specific transcriptional program that generates a hyporesponsive T-cell state [15]. Recently, gliomas have been shown to induce similar phenotypes of T-cell exhaustion [16]. Transcription factors involved in programmed T-cell exhaustion include T-bet, Eomesodermin (Eomes), and NFAT. Exhausted T-cells express high levels of Eomes and low levels of T-bet [17]. While in the exhausted state, failure of NFAT to form a complex with AP-1 results in expression of inhibitory immune checkpoints, such as PD-1 and CTLA-4 [18]. In addition to these conventional ones, other recently characterized checkpoints involved in T-cell exhaustion include TIM-3, LAG-3, BTLA, 2B4, SLAMF6, CD160, TIGIT, and CD39 [3]. A recent study looking at a variety of these exhaustion markers demonstrated that T-cell exhaustion is particularly severe in GBM compared to other types of cancer [16]. The authors showed that co-expression of PD-1, TIM-3, and LAG-3 rendered human GBM tumor-infiltrating lymphocytes (TILs) in a severely hypofunctional state.

The last domain of T-cell dysfunction is T-cell ignorance, which occurs when fully functional T-cells are prevented from antigen exposure by anatomical barriers or insufficient antigen expression levels [19]. Theoretically, ignorance can be overcome by a sufficient quantity of T-cells undergoing antigen exposure. However, GBM patients frequently exhibit clinically significant lymphopenia [20]. A recent study again demonstrated this fact, and was able to show this is at least partially produced by T-cell sequestration in the bone marrow due to the loss of S1P1 receptors from the T-cell surface [20]. Lymphopenia combined with the blood brain barrier (BBB) limiting access into the immunologically-distinct brain prevents the antigen exposure necessary to produce robust, T-cell mediated immune responses in the tumor microenvironment (TME).

#### Regulatory T-cells (Tregs)

**Fig. 2:**
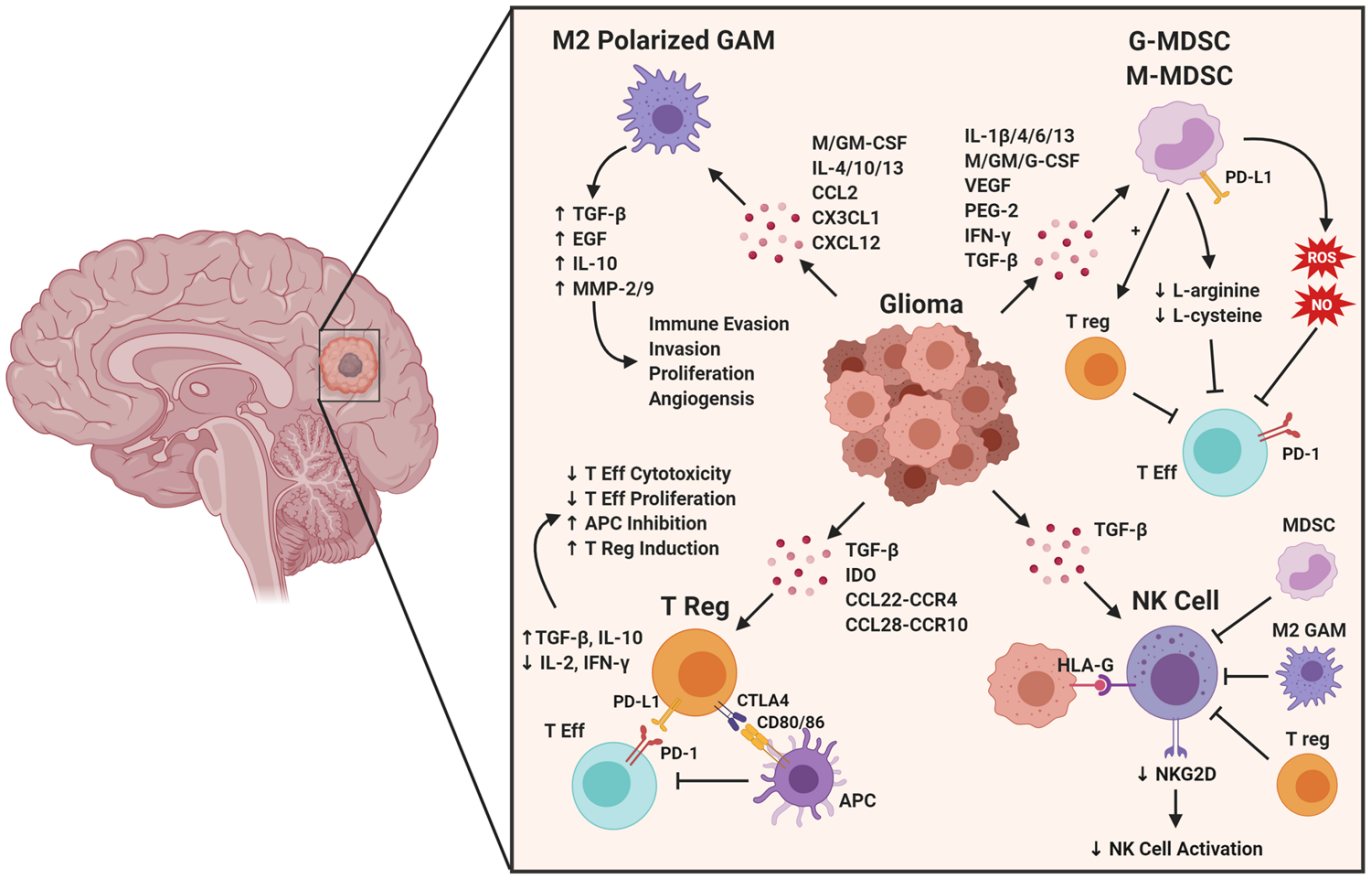
Summary of glioma-immune interactions. Gliomas secrete or express a variety of factors that attract or induce immunosuppressive cell types, or have direct inhibitory effects on immune effector cells. *T Eff* effector T cell, *ROS* reactive oxygen species, *NO* nitric oxide, *GAM* glioma-associated microglia/macrophage, *MDSC* myeloid-derived suppressor cell, *T reg* regulatory T cell, *MHC* major histocompatibility complex, *APC* antigen presenting cell. Created with BioRender.com

Tregs are characterized by their ability to suppress effector T-cell activation through a variety of mechanisms (Fig. 2), most notably secretion of immunosuppressive cytokines and downmodulation of co-stimulatory molecules on antigen presenting cells (APCs) [21]. The glioma TME favors recruitment and survival of Tregs by maintaining high concentrations of cytokines that support Treg persistence, such as transforming growth factor-β (TGF-β) and indoleamine 2,3-dioxygenase (IDO) [22, 23]. While Tregs normally represent 5–10% of circulating CD4^+^T-cells, they are found in increased numbers and frequencies in a multitude of cancers, with higher numbers of Tregs associated with a worse prognosis [24, 25]. Glioma patients have higher proportions of circulating Tregs compared to healthy controls (even though absolute Treg numbers were decreased), and these patients have increased Treg numbers infiltrating the tumors themselves [26, 27]. These findings were recapitulated in murine glioma models, with subsequent studies demonstrating that Treg depletion prolonged survival in glioma-bearing mice [26]. Consequently, novel therapeutic approaches to either inhibit or reduce Treg numbers are an active area of research [27–29].

#### Natural killer (NK) cells

NK cells are innate lymphoid cells capable of directly lysing infected or malignant cells. NK cells can target other cells missing MHC Class I, an adaptive process that is used by many viruses and tumors to evade detection by T-cells [30, 31]. By expressing a combination of inhibitory and stimulatory receptors, NK cells can tailor their response to specific insults [32]. For example, killer cell immunoglobulin-like receptors (KIR) can recognize MHC Class I present on healthy cells, preventing NK cell activation. In contrast, stressed or infected cells upregulate ligands that bind NKG2D, an activating receptor that triggers NK cell-mediated killing of the target cell. The importance of NK cells in cancer is demonstrated by the fact that mice and humans with NK cell deficiencies are at a higher risk to develop certain malignancies [33, 34]. In GBM, some populations of patients have decreased levels of NKG2D on the surface of their NK cells, leading to decreased NK cell activation [35]. Additionally, HLA-G, an inhibitory ligand found on gliomas, is able to bind to NK receptors in the KIR family (such as KIR2DL4 and ILT2) and inhibit NK cytotoxicity, IFN-γ secretion, NKG2D activation, and chemotaxis (Fig. 2) [36]. Despite NK cells making up a relatively small proportion of tumor-infiltrating cells, studies have shown that these NKs residing in the GBM TME display characteristics that allow them to be considerably cytotoxic to tumor cells in other cancers [37]. Therefore, potential therapeutic opportunities are actively being pursued that focus on either modulating NK cell numbers/activation status, or utilizing chimeric antigen receptor (CAR) technology to generate NK cells expressing receptors that specifically target tumor antigens.

### Myeloid dysfunction

#### G/M-MDSCs

Myeloid-derived suppressor cells (MDSCs), identified as CD11b^+^CD33^+^HLA-DR^−/low^ cells, are a heterogeneous population of immature myeloid cells that also play an important role in tumor-induced immunosuppression [38]. MDSCs, whose phenotype comprises 20–30% of the bone marrow, make up only 0.5% of peripheral blood mononuclear cells (PBMCs) as they quickly differentiate into mature subtypes in a normal, non-pathologic state. However, in disease states such as cancer, this population increases significantly due to alterations in myelopoiesis [39]. To date, elevated levels of MDSCs have been found in melanoma, glioma, renal, gastric, bladder, esophageal, and pancreatic cancers [40]. GBM, however, has one of the highest levels of circulating MDSCs of these cancers, with ~ 12 × greater than normal levels [41–43].

MDSCs, whose two major subsets include granulocytic (G-MDSC, identified as CD15^+^ in addition to the previously mentioned markers) and monocytic (M-MDSC, additionally CD14^+^), exert their immunosuppressive effects through inhibition of innate antitumor immunity by several mechanisms (Fig. 2) [44, 45]. These mechanisms include: expression of arginase, which decreases the level of L-arginine in the blood/tumor (an amino acid needed for normal T-cell function, specifically translation of the T-cell CD3 zeta chain); secretion of nitric oxide and production of ROS, which themselves are capable of inducing T-cell suppression; and expression of PD-L1 to participate in checkpoint blockade [46, 47]. Raychudhuri et al*.* demonstrated that T-cells obtained from GBM patients have suppressed IFN-γ production, and that removal of MDSCs from the patients’ PBMC population restored T-cell function [41]. In addition, several other studies have shown secretion of immunosuppressive cytokines, Treg stimulation, and the positive relationship between immunosuppression and tumor angiogenesis, which is mediated by MDSCs and dependent on STAT3 activation [39, 48, 49].

In light of their widespread immunosuppressive effects, elevated levels of MDSCs have been shown to be correlated with clinical cancer stage, histologic tumor grade, metastatic tumor burden, radiographic progression, and/or prognosis in a variety of cancers [46, 50, 51]. While the volume of literature linking MDSCs to these clinical variables in glioma is not as robust as in other types of cancer, recent publications have focused on this topic. Alban et al*.* found that GBM patients with a better prognosis had decreasing numbers in their peripheral circulation over time, as well as reduced MDSCs in their tumors [52]. Another study found that a subtype of G-MDSCs accumulated in the peripheral blood of GBM patients, and correlated with reduced numbers of effector immune cells, early recurrence, and disease progression [53]. In light of these results, a trial was performed in GBM patients to reduce MDSCs in peripheral circulation and increase cytotoxic immune infiltration into the TME [54]. Future studies are needed to further assess the association of MDSCs to clinical disease course.

#### Tumor-associated macrophages/microglia

Tumor-associated macrophages (TAMs) and their resident CNS correlate, microglia, are able to infiltrate gliomas and comprise a substantial proportion of cells in the TME, up to 15–30% depending on glioma grade [55]. While microglia are yolk sac–derived with the capacity for limited self-renewal, TAMs are monocyte-derived from the bone marrow and peripheral circulation, extravasating into the tumor as a result of the breakdown of the BBB near the tumor [56]. While glioma-infiltrating TAMs and microglia (termed glioma-associated microglia/macrophages [GAMs] as a group) have been identified in the past by the markers CD163, CD200, CD204, CD68, and Iba-1, the most common identification strategy in the literature considers microglia to be CD11b^high^CD45^low^, while TAMs are CD11b^high^CD45^high^ [51]. Multiple studies have shown the correlation between the number and morphology of GAMs with glioma grade (higher numbers and amoeboid morphology), as well as increases in GAM numbers correlating with increased aggressiveness within specific tumor grades [57–61].

GAMs are noted to have a significant degree of plasticity in regards to their effector functions. The M1 phenotype is considered pro-inflammatory and anti-tumor, typically acquired after stimulation with GM-CSF, toll-like receptor 4 (TLR4) ligands, and/or IFN-γ [51, 62]. Conversely, the M2 phenotype is considered cytoprotective, immunosuppressive, and protumorigenic, occurring after M-CSF (expressed by glioma cells, as well as normal human astrocytes), IL-4, IL-10 and/or IL-13 exposure. The M2 polarized GAMs produce high levels of IL-10, transforming growth factor (TGF)-β, epithelial growth factor (EGF), matrix metalloproteinase (MMP)-2 and MMP-9, and low levels of IL-12, which overall promotes tumor cell immune evasion, invasion, proliferation and angiogenesis (Fig. 2) [51, 62]. However, it should be noted that these phenotypes were generated in vitro under ideal conditions, and thus GAMs in vivo likely have a variety of functions along the M1/M2 spectrum (moreover, additional subpopulations have also been defined, such as M2a, M2b, M2c, etc.) [55]. Recent work now aims to go beyond cell surface markers to gather in depth gene expression profiling data, to gain greater understanding of the functions of GAMs and discern potential therapeutic targeting strategies [63, 64].

## Tumor-related immunosuppressive factors

### Glioma cell surface factors and cytokine secretion/dysregulation

Gliomas employ several mechanisms to evade the immune system. Among others, these include modulation of cell surface molecules, and secretion of cytokines. Gliomas can express PD-L1, and when bound to PD-1 on T-cells, can suppress T-cell activation. In addition, gliomas downregulate HLA-class I and can upregulate certain HLA-class II molecules, resulting in a deficient cytotoxic T-cell response and skewing toward a CD4^+^T-cell response. Gliomas also have the capacity to interfere with antigen processing or presentation on HLA [65, 66].

Cytokines play an important role in glioma progression, as they can affect proliferation, angiogenesis and aggressiveness of the tumor. Classic immunosuppressive cytokines associated with glioma are TGF-β and IL-10. TGF-β levels are associated with glioma grade, triggering proliferation in HGGs. It is also a regulator of VEGF (vascular endothelial growth factor), implicated in angiogenesis [67]. TGF-β suppresses lymphocytes and NK cells and can cause inhibition of antigen presentation [68]. In addition to TGF-β, IL-10 is largely responsible for shifting the TME toward an immunosuppressive phenotype. IL-10 can be produced from the glioma directly or gliomas can stimulate the production of IL-10 by macrophages and microglia [67, 68]. IL-1β, a classical pro-inflammatory cytokine, is also overexpressed in gliomas as compared to healthy controls, and has been shown to regulate both the survival and invasiveness of GBM. IL-6, TNF-α, and IL-8 have all also been shown to be upregulated in gliomas as compared to healthy individuals and play a role in tumor growth and invasion [69].

### TME hypoxia

Tumor cell viability and response to therapeutic agents is highly influenced by several factors, including tissue hypoxia. Hypoxia, defined as an oxygen saturation of less than 2% (compared to 2–9% in healthy tissue), is a hallmark of the GBM TME [70]. Low oxygen tension (i.e. hypoxia) is caused by the tumor cells rapidly outgrowing their blood and nutrient supply, resulting in increased cellular necrosis and acidosis [71, 72]. Gliomas adapt to the hypoxic TME via oxygen-sensitive transcription factors called hypoxia-inducible factors (HIFs), the most notable of them being HIF-1α and HIF-2α [72]. These HIFs play an important role in tumor growth and survival through regulation of several key components of tumor biology, including glycolytic metabolism, pH homeostasis, angiogenesis, mitochondrial autophagy and resistance to apoptosis [72, 73].

HIF activation is also important for tumor immunogenicity, as certain immune cells that promote tumorigenesis can infiltrate and preferentially target these areas of hypoxia [72, 74]. TAMs have been shown to infiltrate hypoxic regions within solid tumors, with VEGF increasing TAM recruitment in a HIF-dependent manner [72, 74]. Likewise, tumor-associated fibroblast expression of the chemoattractant CXCL12 is upregulated under hypoxic conditions and also plays an important role in TAM recruitment [72]. While TAM polarization in the M1 or M2 phenotype is mainly induced by interferon-regulatory factor/signal transducer and activator of transcription (IRF/STAT) signaling pathways, hypoxia also can regulate this phenomena and activate HIFs differently to induce an M1 or M2-like phenotype [75]. Specifically, HIF2α activation is involved in the M2 polarization axis, with these TAMs being associated with immunosuppression, tumor cell proliferation, angiogenesis, and local invasion, resulting in poor patient outcomes [76, 77]. Similarly, elevated expression of HIF-2α is associated with poor prognosis and higher tumor grade in numerous cancer types [78]. Due to these reasons, HIFs may be a promising treatment target, with studies in several murine models showing that HIF inhibition (e.g. acriflavine) improves destruction of cancer cells and increases survival [73].

## Systemic/treatment-related immune suppression

### Steroid therapy

The use of high-dose glucocorticoids, such as dexamethasone, is standard of care to reduce the life-threatening vasogenic edema seen in patients with CNS tumors. Although the exact mechanism is not well understood, several studies have proposed that glucocorticoids reduce cerebral edema by stabilizing the capillary membrane and blocking expression of VEGF [79, 80]. However, the potent anti-inflammatory and immunomodulatory effects of dexamethasone are well described in the literature, producing clinically significant lymphopenia via signaling through the lymphotoxic glucocorticoid receptors on both B and T lymphocytes, and attenuating the CD28 co-stimulatory pathway [81, 82]. Studies have shown that dexamethasone doses as little as 0.25 mg/kg/day result in reduced numbers of TILs and other important immune cells in the TME [83]. Therefore, the positive benefits of edema reduction are countered by the negative sequelae of immune suppression. While steroid administration is an absolute necessity in many circumstances, their immunosuppressive side effects should prompt dose reduction or cessation by clinicians whenever possible, especially in patients that are on immunotherapies.

### Chemotherapy

Glioma patients may be repeatedly pancytopenic for periods of time due to chemotherapy-induced myelosuppression and myeloablation, exposing them to the risk of infection and limiting mechanisms of innate anti-tumor immunity (Table 1). The most commonly used chemotherapeutic in glioma treatment is temozolomide, a DNA methylator that is known to cause long-lasting lymphopenia [84, 85]. Additionally, the use of temozolomide is associated with an upregulation of T-cell exhaustion markers such as LAG-3 and TIM-3, which has unique implications for its concomitant use with immunotherapy [86]. As studies have shown that treatment-related immunosuppression from temozolomide/radiation therapy is long-lasting and associated with early death from tumor progression in HGG patients, new approaches need to be devised to overcome these detrimental effects [85]. Recent work by Karachi et al*.* demonstrated that metronomic dosing of temozolomide in combination with anti-PD-1 therapy decreased TIL exhaustion markers and rescued the survival benefit seen with immunotherapy in two syngeneic murine GBM models. As temozolomide is part of the current standard of care treatment of GBM, further evaluation of this study and others is needed [86].

**Table 1:** Chemotherapeutic drugs commonly used alone or in combination for the treatment of malignant tumors of the CNS

Chemotherapeutic^a^	Mechanism of action^b^	Myelosuppression score^c,d^
Carmustine/Lomustine	DNA cross-linking/alkylating agent	4
Carboplatin	DNA cross-linking/alkylating agent	3
Cisplatin	DNA cross-linking/alkylating agent	1
Cyclophosphamide	DNA cross-linking/alkylating agent	3–4 (based on dose)
Etoposide	DNA Topoisomerase II inhibitor	4
Irinotecan	DNA Topoisomerase I inhibitor	4
Methotrexate	Anti-metabolite (dihydrofolate reductase inhibitor)	2
Procarbazine	DNA cross-linking/alkylating agent	Unavailable
Temozolomide	DNA cross-linking/alkylating agent	2
Vinblastine	Cell cycle specific microtubule/tubulin inhibition	2
Vincristine	Cell cycle specific microtubule/tubulin inhibition	0

^a^National Comprehensive Cancer Network. NCCN Clinical Practice Guidelines in Oncology: Central Nervous System Cancers. V.2.2019. Accessed at www.nccn.org/professionals/physician_gls/pdf/cns.pdf on October 5, 2019

^b^Lexicomp Online, Hudson, Ohio: Wolters Kluwer Clinical Drug Information, Inc.; 2013; January 28, 2020

^c^Lalami Y, Paesmans M, Muanza F, et al. (2006) Can we predict the duration of chemotherapy-induced neutropenia in febrile neutropenic patients, focusing on regimen-specific risk factors? A retrospective analysis. Ann. Oncol, 17:507–514. 10.1093/annonc/mdj092

^d^Based upon single drug therapy. A weight (0–4) is assigned to each drug according to its expected frequency of severe neutropenia (0 unusual, 1 very rare, 2 rare, 3 frequent, 4 very frequent)

While these negative chemotherapy-induced side effects are well noted and should be minimized whenever possible, a recently-devised strategy uses the lymphotoxicity of temozolomide to the clinician’s advantage within a specific treatment paradigm. Suryadevara and colleagues were able to utilize a dose-intensified temozolomide regimen to deplete host lymphocytes prior to CAR administration, which was associated with dramatically improved CAR proliferation, complete tumor regression, and increased survival in a murine model of GBM [84]. Examples such as this one highlight the ability of clinicians and researchers to develop innovative and/or unconventional uses of traditional chemotherapeutics to enhance antitumor immunity.

## Conclusions

Gliomas create a profoundly immunosuppressive environment both locally at the tumor and systemically in the body, creating a number of challenges that negatively impact patient well-being and efficacy of novel immunotherapeutic approaches. In attempting to understand the pathobiology of these complex tumors, a multitude of mechanisms have been uncovered by which neoplastic cells develop the ability to evade detection and destruction by the immune system. By targeting one or more of these mechanisms, researchers hope to discover the next major treatment breakthrough that provides a meaningful survival benefit to a patient population greatly in need of one.
